# New Directions in Cariology Research 2011

**DOI:** 10.1155/2012/392730

**Published:** 2012-03-01

**Authors:** Alexandre R. Vieira

**Affiliations:** Department of Oral Biology, School of Dental Medicine, University of Pittsburgh, Pittsburgh, PA, USA

Due to the great success of the “New Directions in Cariology Research” first issue, we decided to repeat the experience and here you have the “New Directions of Cariology Research 2011.” This issue coincides with the disturbing record of another death in the United States, a 24-year-old male from Cincinnati, OH, consequence of untreated carious lesions. It is still a puzzle to many how a largely preventable disease remains highly prevalent, affecting 80% of the people alive in the globe. 

This special issue is a sample of the current research efforts addressing issues related to the pathogenesis of caries. I would like to thank the authors for their excellent contributions, in addition to the many colleagues who assisted in the peer-review process. Finally, I would like to thank the support provided by my guest editors, Dr. F. Seymen, from the Istanbul University, Turkey and Dr. M. Buzalaf, from the University of São Paulo, Brazil.

Aspects related to the disease presentation can be appreciated in Grimoud et al., Honkala et al., Y. Kawashita et al., and Jindal et al. Caries is dependent on biofilm formation, and T. Hart et al., E. Decker et al., Tanaka et al., and M. Bockmann et al. are examples of current approaches to better understand the dynamic relationship between bacterial colonization and the host. How fluoride in the oral cavity environment interferes in the process of lesion formation continues to be of interest and, Chow et al. further explore this topic. Finally, genetics susceptibility to caries is an emerging area of interest in cariology, and D. Gati and A. Vieira, J. Brancher et al., and M. Levine provide examples of the challenges faced by this line of work.

I invite you to read, evaluate, and share this collection of 12 papers that comprise this 2011 Special Issue. Furthermore, I hope the readers will be interested in participating more actively in this debate of what approaches are more efficient to revert the current figures of caries prevalence, and what aspects of this disease should be the focus of research in the coming years.

*Alexandre R. Vieira* *Alexandre R. Vieira*

